# Is Canada ready for patient accessible electronic health records? A national scan

**DOI:** 10.1186/1472-6947-8-33

**Published:** 2008-07-24

**Authors:** Sara Urowitz, David Wiljer, Emma Apatu, Gunther Eysenbach, Claudette DeLenardo, Tamara Harth, Howard Pai, Kevin J Leonard

**Affiliations:** 1 Princess Margaret Hospital/University Health Network, Canada; 2 Department of Radiation Oncology, University of Toronto, Canada; 3 Department of Health Policy, Management and Evaluation, University of Toronto, Canada; 4 Centre for Global eHealth Innovation, University Health Network, Canada; 5 Grand River Hospital, Canada; 6 Odette Cancer Centre/Sunnybrook Health Sciences Centre, Canada; 7 Department of Surgery, University of British Columbia, Canada; 8 School of Health Information Sciences, University of Victoria, Canada; 9 BC Cancer Agency, Victoria, Canada

## Abstract

**Background:**

Access to personal health information through the electronic health record (EHR) is an innovative means to enable people to be active participants in their own health care. Currently this is not an available option for consumers of health. The absence of a key technology, the EHR, is a significant obstacle to providing patient accessible electronic records. To assess the readiness for the implementation and adoption of EHRs in Canada, a national scan was conducted to determine organizational readiness and willingness for patient accessible electronic records.

**Methods:**

A survey was conducted of Chief Executive Officers (CEOs) of Canadian public and acute care hospitals.

**Results:**

Two hundred thirteen emails were sent to CEOs of Canadian general and acute care hospitals, with a 39% response rate. Over half (54.2%) of hospitals had some sort of EHR, but few had a record that was predominately electronic. Financial resources were identified as the most important barrier to providing patients access to their EHR and there was a divergence in perceptions from healthcare providers and what they thought patients would want in terms of access to the EHR, with providers being less willing to provide access and patients desire for greater access to the full record.

**Conclusion:**

As the use of EHRs becomes more commonplace, organizations should explore the possibility of responding to patient needs for clinical information by providing access to their EHR. The best way to achieve this is still being debated.

## Background

E-Health is described as a revolutionary new paradigm for health care that has evolved as a result of advances in information, telecommunication, network technologies and information management. These technologies have transformed the way that health care is delivered [1,2]. Today's technology has the capability to support people in managing almost all aspects of their health care, from seeking general health information to clinical consults without ever having to leave their homes; yet even the most basic personal health information, like specific results of tests, contained in medical charts is not currently readily available through existing technologies (like the Internet) to most consumers of health care [3,4]. This inaccessibility makes it difficult for consumers of health care to be active participants in their own health and wellness. There is consensus that in order for patients to be true partners in the health care encounter, they must have access to their own personal clinical health information (Wiljer et al., Patient Accessible EHRs: Building consensus for successful implementation strategies, *submitted*) that is commonly stored in institutional electronic health records (EHRs). An Electronic Health Record (EHR) is a computerized version of an individual's health record that may contain a person's full health and medical record or can be used for certain records, such as lab results, in conjunction with a more traditional paper-based patient chart. The EHR may be accessible online from many separate, interoperable automated systems within an electronic network and it can facilitate the electronic integration of health care providers by enabling the retrieval of information about patients when and where it is most needed [5]. The ability to provide patients with access to their personal health information can be facilitated through the use of emerging technologies, most commonly through the Internet [6-9]. This type of access to one's own health information can help prepare individuals to better manage and cope with their health status. In turn, this type of access may have a positive impact on the health care system that can be recognized through more efficient use of resources resulting in health care savings [3]. Access to personal health information is a fundamental right supported by the law [10] and the emergence of new technologies alters how that right can be fulfilled. It has been demonstrated that access to one's health information using these technologies is desired by many health care consumers [7,11-15].

Early adopters of new technologies in health care, primarily in the United States and the United Kingdom, are reporting the potential benefits of providing people with access to their electronic health record [6,9,13,14,16-18], with little to no disruption in clinical operations [19]. Access to one's medical record in either traditional paper format or electronically can potentially: 1) enhance patients' understanding of their condition [20]; 2) empower individuals to become active participants in their own care [21-23]; 3) result in better medical management [24,25]; 4) lead to more effective provider-patient communication [20,25-27] and; 5) may improve satisfaction and outcomes [25,28], possibly through improved adherence to health promotion recommendations [29]. A recent randomized trial (ClinicalTrials.gov: NCT00142077) demonstrated a modest impact of a Personal Health Record-based employee health program with tailored, targeted health messages on influenza prevention and control [30]. Providing patient access to their personal health information is also cited as a way of achieving a model of patient-centred care [31].

Although technology exists to support patient access to their medical record, and the law supports individuals' rights to access their health record the health care community has been slow to adopt the use of patient accessible electronic health records. Although several reasons could be cited for this slow uptake process, the absence of a key technology, the EHR, is a significant obstacle to making progress in providing patient accessible electronic records. A 2003 white paper from the American Association of Medical Informatics identifies "the lack of ubiquitous EHR usage" as the main environmental barrier to patient accessible health records [32].

There has recently been a noticeable increase in institutional interest in and adoption of EHRs and Canada Health Infoway has set the target of 50% of Canadians having their electronic health records available to their healthcare providers by 2010 [33]. The adoption of systems that provide patient access to these EHRs, such as patient portals or personal health records (PHRs) has been slower to follow. Systems such as these capture either elements of data or all the data stored in the EHR [5] and can be easily provided to the consumer. A PHR system can also incorporate data entered by patients themselves. Slower growth in this area may result from physicians' reluctance to embrace the use of information and communication technology (ICT) solutions [34-36].

To assess the readiness for the implementation and adoption of EHRs and PHRs in Canada, a national scan of Canadian general and acute care hospitals was conducted to determine organizational readiness for patient accessible records, to understand organization and staff values related to patient access to their records, and organizational perceptions of patients desires and needs. The scan was conducted under the auspices of the Canadian Committee for Patient Accessible Electronic Health Records (CCPAEHR). The CCPAEHR is a group of Canadian researchers, clinicians, information specialists and educators working together to assess and promote patient access to and involvement with EHRs. This paper will report on the results of this scan and will draw conclusions in an attempt to elucidate the trends in the adoption of EHRs in Canadian hospitals, and the movement towards adoption of PHRs and patient accessible EHRs.

## Methods

### Study population

The frame sample was generated from Scott's Canadian Medical Directory. Eligibility required a designation of either public or acute care hospital, and an active email address for the institutions' Chief Executive Officer (CEO).

### Research design

Based on a review of the literature and input from members of CCPAEHR and staff at Canada Health Infoway, a questionnaire was created to measure national readiness for the adoption and implementation of the EHR and perceptions regarding the use of EHR and PHR [See Additional file 1]. The survey instrument included questions pertaining to current record keeping practices using a paper based record and an EHR, the information technology infrastructure to support the EHR, and perceptions about providing patient access to the EHR. Access was defined broadly and could include a number of solutions. For the purpose of this study, we were interested in any configuration of electronic patient information that could be called an EHR, including a computerized record of a person's health and/or medical history that may contain a person's full health and medical record, or just certain records, such as laboratory or diagnostic testing results. Having access to these results provides people, particularly those with chronic illness, access to important information that can help in disease self-management. The questionnaire was reviewed, and tested for face validity by members of the CCPAEHR.

Data collection took place over an eight-week period and CEOs of eligible institutions were emailed a letter of introduction and a link to the electronic questionnaire in English and French. The CEO was asked to complete the questionnaire and submit it within two weeks of receipt. In addition, the CEO was asked to forward the link for the questionnaire to the chiefs of medicine, nursing and informatics, or the individual who was regarded as the most appropriate to respond. A reminder email message was sent two weeks after initial contact was made, and again, three weeks prior to the close of study date.

### Analytic approach

Data were analyzed using both Questionpro [37] and the SPSS statistical analysis package. Descriptive statistics were used to summarize the basic features of the data, and cross tabulations were used to show the relationship between variables.

### Ethics

This study was approved by the Research Ethics Board (06-0318-CE) of the University Health Network.

## Results

Two hundred thirteen emails were sent to CEOs of Canadian general and acute care hospitals with a 39% (83/213) response rate.

### Demographics

As shown in Table 1, while the majority of respondents were from Ontario 58.8% (n = 30), there was representation from across the country. Most of the respondents (67.9%) selected "other" to describe their role. These individuals most commonly self identified as either Managers of Health Records or Health Information Services or Privacy Officers. Just under half of the responses came from institutions with fewer than 100 beds, but there were responses from a number of medium-sized and larger hospitals as well. One respondent answered on behalf of a health care system (which included 13 hospitals in total), but over half (58.5%) indicated that their hospital was part of a larger health care system.

**Table 1:** Demographics of Responders to Survey

**Demographic**		**Respondents %**
**Location**	Ontario	58
**N = 50**	British Columbia	16
	Manitoba	10
	Alberta	8
	North West Territories	4
	New Brunswick	2
	Nunavut	2

**Role**	Chief Executive Officer	9.4
**N = 54**	Chief of Medicine	3.8
	Chief of Nursing	11.3
	Chief Information Officer	7.6
	Other	67.9*

**Hospital size**	Less than 100	49.0
**N = 51**	100 to 400	33.3
	More than 400	17.7

*Other roles most commonly included Managers of Health Records, Health Information Services or Privacy Officers.

### The electronic record

Just over half (54.2%) of hospitals surveyed reported having some sort of EHR; however, 97.6% indicated that the EHR was not the sole method for recording patient information. There were very few institutions that had predominately an electronic record; most commonly (39%) hospitals had records that were between 11–50% electronic (Table 2).

**Table 2:** Percent of Patient Record that is Electronic N = 41

**% Electronic**	**Respondents %**
0–10%	29.3
11–50%	39
51–90%	29.3
91–100%	2.4

Almost half (44.6%) of the respondents thought that their institution was "on track" with the rest of the country in terms of adoption and use of an EHR; 35.4% indicated that their institution was lagging behind on adoption and implementation. For hospitals that have an electronic record, adoption of the EHR was most commonly a recent phenomenon. Just over half (67.5%) of respondents reported adoption of the EHR within the last 5 years (Table 3).

**Table 3:** Time from Commencement of Adoption of EHR N = 43

**Years**	**Respondents %**
<1 yr	16.3
>1 yr	51.2
>5 yrs	27.9
>10 yrs	4.7

### Perceptions about providing access to the EHR

Survey respondents identified hospital financial resources as the most important barrier (86.7%) to effectively providing patients access to their EHR. Patient computer literacy and clinician buy-in were also thought to be very important barriers to patient access (Table 4).

**Table 4:** Importance of Barriers to PAEHRs N = 54

**Barriers**	**Very Important %**	**Important %**	**Moderately %**	**Not Important %**	**Not Very Important %**
Finances	66.7	9.2	16.7	3.7	3.7
Clinician buy-in	50	27.8	14.8	7.4	0
Patient Access to Computers	27.8	22.2	33.3	11.1	5.6
Patient Computer Literacy	48.1	13	22.2	9.3	7.4

Only 3% of respondents had conducted any formal survey of staff perceptions about providing patients with access to their EHR and there were no respondents who had conducted a survey of patient perceptions regarding access to their EHR. Very few (3.6%) respondents thought that staff would be willing and eager to provide patient access to their EHR. Just over one-quarter (28.6%) thought that staff would be hesitant but willing to provide such access and 17.9% thought that staff would support partial access for patients to their EHR. There were an additional 50% who responded that staff perceptions were unknown to them, or that no survey had been conducted. Respondents were also asked to give their opinions on what they thought patients' perceptions were regarding access to their EHR. Less than one half of respondents thought that patients would be eager (17%) or hesitant (17%) to access their electronic health record.

When asked about which elements of the EHR people should have access to, the respondents indicated that staff would be most willing to provide patients with access to their test results (25%) and diagnosis (20.2%), but a quarter thought that patients would desire access to their full record (Table 5).

**Table 5:** Respondents' Perceptions of Provider and Patient Attitudes Regarding Accessible Elements of the EHR

**Elements of the EHR**	**Provider % ** **N = 84**	**Patient % ** **N = 68**
Full Record	10.7	25
Tests Results	25	16.2
Diagnosis	20.2	13.2
Pathology Reports	10.7	5.9
Clinician Notes	2.4	2.9

## Discussion

The Canadian health care system is characterized by two trends: the emergence of e-health [1,38] and a shift from paternalistic-type medicine to a consumer-based approach [11,39]. Today's patients can be well versed in their disease, seek information from numerous and varying sources including the Internet and have the desire to be active participants in making health care decisions [40]. Patients are now more commonly regarded as partners in their care [41-44]. They are often eager to retrieve quality health related information on the Internet. There is a growing interest in developing innovative ways of providing access to one's personal health and medical record [6,9,13,16,19,45,46].

The results of this research suggest that Canadian acute care and public hospitals are moving in the direction of adopting EHRs as is indicated in Tables 2 and 3. The results suggest this is especially true in the province of Ontario from which a majority of respondents came. The high percentage of respondents from Ontario reflects a system of less centralization in Ontario than in others provinces across the country. Over half of the respondents to this study had some sort of EHR in place for between 1 – 5 years, but the EHR was not the only mechanism for recording patient data, as shown in Table 2.

A significant number of our respondents thought that their institution was on track with the rest of the country in terms of adoption of EHR. This finding supports the general trend towards adoption of this technology; however, with the national agenda of having a fully interoperable pan-Canada EHR by 2010 [47], it is somewhat discouraging that over 30% of institutions self-identified as being behind on adoption and implementation of EHRs. Respondents identified financial barriers as the major obstacle to implementation of EHRs. This result reflects the perception of respondents; not necessarily the percentage of the institutional budget spent on information technologies (IT); information that was not solicited in this survey.

Organizations seem to be responding even more slowly to the consumerist trend in health care, and people's desire for access to their health information. Less than 25% of participants responded that patients would like access to their full electronic health record and only 16% thought that patients would like access to their lab results. This perception is in contrast to other Canadian studies that suggest that the majority of patients and the public would like access to components of their health record [15]. Although less than 10% of respondents thought that health care professionals would want patients accessing their full EHR, 25% did indicate that they thought patients should have access to some records such as laboratory test results

For successful wide scale adoption of new technologies like EHR, this survey highlights the need for a culture shift in the health care environment to one that better supports embracing new technologies. There were a small number of respondents who self-identified as leaders in Canada in the field of EHR. These early adopters play an important role in influencing and encouraging others in the change process. Early adopters of PHR technologies in the United States and United Kingdom, for example, have reported that the majority of participants found that accessing their health record was easy and that their medical record was complete and accurate [13,14]. The majority of participants in that study found the information in their PHR to be understandable. Only a few respondents were concerned about confidentiality or about the possibility of learning of negative test results [46]. These results suggest that providing people with access to their EHR is potentially less of a problem than is feared by many health care providers. It also suggests that our respondents' perceptions about patient attitudes regarding access to their PHR may not reflect what patients really want.

Our results suggest that administrators of Canadian Healthcare institutions and health care providers are still anxious about providing access to their EHR. When asked about providers' willingness to provide patient access to the EHR only very few respondents thought that providers would be eager and willing to open the record. On the other hand, when asked about patient desire to access the EHR many more respondents thought that patients would be willing and eager. Similarly, in the opinion of the health care administrators and providers, clinicians were less likely to be willing to open up the full EHR, despite the belief that patients would want access to the full record. These results are representative of the disconnect that exists between consumer desire and provider willingness. These results most likely reflect unwillingness on the part of the providers to give up "ownership" (a legal concept) of the medical record. Providers traditionally have seen the record as existing in their domain and have not fully embraced the role of custodian of the record (Wiljer D, Urowitz S, Carter A, Leonard K, Catton P. Guardians and Gatekeepers: Whose Record is it Anyways? *Submitted*). Recognizing this, systems can be implemented that would reduce provider/intuitional hesitation for providing patient access to the EHR. Firstly, having a mechanism in place to deal with patient anxieties that may result from viewing their record is necessary, and secondly there needs to be a refocusing of attitudes related to the understanding of EHR ownership. Traditionally, the perception has been that ownership of the record has resided with the provider or the institution [48], when in fact certain jurisdictions have described the provider/institution more as the custodian of information (PHIPA 54.1). A landmark ruling from the Supreme Court of Canada in 1992 (McInerney vs MacDonald) specified that patients have a right to access their personal health information. Embracing these types of changes would advance the system towards wider adoption of a ubiquitous EHR, which would in turn support readily accessible PHRs.

### Limitations

Due to the complex methodology for distributing the questionnaire to the broadest pool of respondents, the reported response rate may not be completely accurate. Two hundred thirteen emails were sent to CEOs of Canadian general and acute care hospitals who were asked to forward the questionnaire to others in their institutions. CEOs who were responsible for more than one hospital within a health care system only received one link to the questionnaire. There was no method for tracking the number of questionnaires that were forwarded to multiple recipients within each institution, and therefore it was not possible to calculate the actual number of surveys distributed. There were at least 3 hospitals that had multiple respondents and one respondent who completed a single questionnaire for 13 separate hospitals. Although it is possible that more than the original 213 questionnaires were distributed, we can only report an approximate response rate of 39% (83/213) calculated based on the number of surveys originally distributed and the number of unique responses returned.

As a result of the low response rate, the results from this cross sectional survey may not be representative of all Canadian acute care and general hospitals. The low number of respondents to the survey limited the authors to a descriptive analysis without making statistical inferences on the reliability of the comparisons.

## Conclusion

A necessary pre-request for PHR adoption is the availability of EHR solutions. This study was undertaken to determine the readiness of Canadian hospitals to support patient access to the EHR. Readiness was determined not only based on the availability of an EHR but on institutional and provider perceptions of patients' desires, ability and willingness to access their health record.

Results of this study suggests that Canadian hospitals are slowly moving in the direction of patient accessible EHRs. Although 54% of Canadian hospitals have adopted the use of EHRs, this adoption process is still in its infancy. Most institutions are in a state of transition and still have a significant percentage of their records in paper-based format. A full scale adoption of these technologies poses several potential challenges and a significant proportion of Canadian hospitals are not fully prepared or engaged for the implementation of patient accessible EHRs.

The best way to achieve a balance between patients' desire for access to their records and providers' hesitations about providing that access is still being debated. Before widespread use of PHR can become a reality, it is paramount that change occurs in organizational culture around such issues as ownership and rights to personal health information. This requirement for change presents numerous future opportunities for a number of organizational and research projects aimed at addressing these needs. The challenges should not arrest the movement towards the implementation of PHRs, but should fuel research to create the best possible service to meet all of the stakeholders' needs.

## Competing interests

The authors declare that they have no competing interests.

## Authors' contributions

SU participated in the design of the study, the development of the questionnaire, the analysis of data and drafted the final manuscript. DW participated in the design of the study, the development of the questionnaire and contributed to the final manuscript. EA was responsible for data management and contributed to the analysis. GE contributed to the creation of the questionnaire and to the final manuscript. CDL contributed to the creation of the questionnaire and to the final manuscript. TH contributed to the creation of the questionnaire and to the final manuscript. HP contributed to the creation of the questionnaire and to the final manuscript. KJL contributed to the creation of the questionnaire and to the final manuscript.

## Pre-publication history

The pre-publication history for this paper can be accessed here:



## Supplementary Material

Additional file 1 Appendix A. CCPAEHR Survey (English). Click here for file
