# Supplementary material for: Is Canada ready for patient accessible electronic health records? A national scan
Source: BMC Med Inform Decis Mak. 2008 Jul 24;8:33. doi: 10.1186/1472-6947-8-33 (PMC2532996; doi:10.1186/1472-6947-8-33)
Supplement: Additional file 1 — Appendix A. CCPAEHR Survey (English). [file 1472-6947-8-33-S1.doc]

Appendix A:

CCPAEHR Survey (English)

Section I: Paper Based Patient Record

The following questions relate to providing patients with access to their paper based health and medical records.

Does your hospital use a paper based patient record?

- ❑ Yes ❑ No

Are patients permitted to access their paper record?

- ❑ Yes ❑ No

What is the procedure a patient must follow in order to gain access to his/her paper based record?

- ❑ Request a copy from treating physician
- ❑ Request a copy from the medical records department/Health Information

Management department

- ❑ Submit a formal written request to hospital administration

❑ Other

When patients request access to their paper based record how long does it take to fill the request?

- ❑ Immediately
- ❑ Less then one day
- ❑ Between 1 – 3 days
- ❑ Between 4 –7 days
- ❑ Longer than one week
  - 1. ❑ Other ___________________________________

Are patients charged a fee when they request access to their paper based record?

- ❑ Yes
- ❑ No

How much does it cost?

- - 1. ❑ ___________________________________

When patients request access to their paper based record how is the record presented?

- ❑ Full record
- ❑ Summaries only
- ❑ Requested sections only
  - 1. ❑ Other ___________________________________

On average, how many patients request access to their paper based record each month?

- ❑ 1 – 5
- ❑ 5 – 20
- ❑ 20 – 50
- ❑ 50 – 100
  - 1. ❑ more than 100

Are family members permitted to access the paper record?

- ❑ Yes
  - 1. ❑ No

What is the procedure a family member must follow to access the paper record?

- ❑ With written consent from patient
- ❑ Acting as the patient’s guardian
- ❑ Acting with a Power of Attorney
- ❑ Other (describe) __________________________________________________

Section II: The Electronic Health Record

Does your hospital have an electronic health record/electronic medical record/electronic patient record (EHR)?

- ❑ Yes
  - 1. ❑ No

When did your institution adopt the use of an EHR?

- ❑ Within the last 3 months
- ❑ Within the last 6 months
- ❑ With in the last 12 months
- ❑ More than one year ago
- ❑ More than 5 years ago
  - 1. ❑ More than 10 years ago

Where does the funding come from to support the use of the EHR?

- ❑ Your institution
- ❑ Private Industry
- ❑ Research Grants
- ❑ Donations
- ❑ Provincial funds
  - 1. ❑ National funds

Is the EHR the only method for recording patient information?

- ❑ Yes
  - 1. ❑ No

What percentage of the patient record is electronic?

- ❑ 0 - 10%
- ❑ 11 – 50%
- ❑ 51 – 90%
  - 1. ❑ 91 – 100%

Does your institution use an “off the shelf” EHR product?

- ❑ Yes
  - 1. ❑ No

Which of the following describes your EHR? (select all that apply)

- ❑ Customized off the shelf product
- ❑ Built in-house
- ❑ Outsourced
- ❑ Design input from clinicians
- ❑ Design input from patients
- ❑ Collaborative project between several organizations
  - 1. ❑ Reflects format/design of paper chart

Who can make entries into the EHR?

- ❑ Physicians
- ❑ Nurses
- ❑ Allied health care professionals
- ❑ Chaplains
- ❑ Administrative personnel
- ❑ Support staff
- ❑ Community physicians
- ❑ Patients
- ❑ Family members
- ❑ Other ___________________________________

We are interested in learning about ownership of and access to the EHR.

In your hospital who is identified as the official owner of the EHR?

- ❑ Hospital
- ❑ Health care system
- ❑ Health care provider
- ❑ Patient
  - 1. ❑ Other ___________________________________

In your hospital who is permitted to access the EHR without special authorization? (Select all that apply)

- ❑ Physicians
- ❑ Nurses
- ❑ Allied health care professionals
- ❑ Chaplains
- ❑ Administrative personnel
- ❑ Support staff
- ❑ Community physicians
- ❑ Patients
- ❑ Family members
  - 1. ❑ Other ___________________________________

What is the procedure a patient must follow in order to gain access to his/her EHR?

- ❑ Request access from the treating physician
- ❑ Request access from the medical records department/Health Information

Management department

- ❑ Submit a formal written request to hospital administration
- ❑ Other ____________________________________________________________

In order to access the EHR online are there any other procedures that patients must follow (select all that apply)?

❑ Obtain a password

❑ Provide official consent

❑ Complete training

❑ None

❑ Other ___________________________________

What is the average yearly cost of providing patients with access to their EHR? (List in Canadian Dollars)

❑ ___________________________________

On average, how many patients request access to their EHR each month?

❑ 1 – 5

❑ 5 – 20

❑ 20 – 50

❑ 50 – 100

❑ More than 100

The following questions pertain to policies related to the EHR.

Is it a written institutional policy that all patient information be record in the EHR?

- ❑ Yes
  1. ❑ No

Would you be willing to provide a copy of the policy?

- ❑ Yes
- ❑ No

Please mail or email a copy of the policy to:Dr. Sara Urowitz, Research Associate and Postdoctoral Fellow, Oncology Education and Survivorship, University Health Network Princess Margaret Hospital 14-411610 University Ave. Toronto, ON M5G 2M9sara.urowitz@uhn.on.ca

Does your hospital have a written policy on providing patients with access to the EHR?

- ❑ Yes
  1. ❑ No

Would you be willing to provide us with a copy of the policy?

- ❑ Yes
- ❑ No

Please mail or email a copy of the policy to:Dr. Sara Urowitz, Research Associate and Postdoctoral Fellow, Oncology Education and Survivorship, University Health Network Princess Margaret Hospital 14-411610 University Ave. Toronto, ON M5G 2M9sara.urowitz@uhn.on.ca

Does your hospital have an informal policy on providing patients with access to the EHR?

- ❑ Yes
- ❑ No

Please describe

❑ ___________________________________

Does your hospital have a policy on maintaining confidentiality of the EHR?

- ❑ Yes
  1. ❑ No

Would you be willing to provide us with a copy of the policy?

- ❑ Yes
- ❑ No

Please mail or email a copy of the policy to: Dr. Sara Urowitz, Research Associate and Postdoctoral Fellow, Oncology Education and Survivorship, University Health Network, Princess Margaret Hospital 14-411610 University Ave. Toronto, ON M5G 2M9sara.urowitz@uhn.on.ca

What do you perceive are the key factors in driving faster adoption of EHR solutions?

|  |  | Least Important |  |  | Somewhat Important |  |  | Most Important |
| --- | --- | --- | --- | --- | --- | --- | --- | --- |
| Consumer demand |  |  |  |  |  |  |  |  |
| Provider demand |  |  |  |  |  |  |  |  |
| Executive leadership |  |  |  |  |  |  |  |  |
| Access to funding |  |  |  |  |  |  |  |  |
| Access to better information |  |  |  |  |  |  |  |  |
| Other |  |  |  |  |  |  |  |  |

Please describe any Other key factors What do you perceive are the key barriers in developing or implementing an EHR?

❑ ___________________________________

|  | Least Important |  | Somewhat Important |  | Most Important |
| --- | --- | --- | --- | --- | --- |
| Cost |  |  |  |  |  |
| Privacy concerns |  |  |  |  |  |
| Coordination of multiple stakeholders across continuum of care (e.g. governance) |  |  |  |  |  |
| Education and training about EHR |  |  |  |  |  |
| Time and effort required to develop or implement |  |  |  |  |  |
| Other |  |  |  |  |  |

Please describe any other barriers

❑ ___________________________________

There are several key benefits for providing an EHR. In order of priority what do you perceive are the key benefits an EHR provides?

|  | Least  Important |  | Somewhat Import ant |  | Most Imp ortant |
| --- | --- | --- | --- | --- | --- |
| Patient safety |  |  |  |  |  |
| Clinical workflow efficiencies |  |  |  |  |  |
| Quality of care |  |  |  |  |  |
| Continuity of care |  |  |  |  |  |
| Other |  |  |  |  |  |

Please describe any Other benefits

❑ ___________________________________

With respect to other EHR initiatives in Canada, where do you think your organization ranks?

- ❑ LaggingBehind
- ❑ On TrackWith Others
- ❑ Leader

Section III: The Information Technology Infrastructure

We are interested in learning about your institution’s current information technology (IT) infrastructure and its ability for supporting access to the EHR. (Please answer to the best of your ability even if no EHR is used.)

Does your current IT infrastructure support the use of an EHR?

- ❑ Yes
  - 1. ❑ No

Does your current IT infrastructure support patient access to the EHR?

- ❑ Yes
  - 1. ❑ No

Are there plans for implementing appropriate IT infrastructure?

- ❑ Yes
- ❑ No

Please explain why not. Please describe

❑ ___________________________________

From which locations can patients access their EHR?

- ❑ Hospital computer
- ❑ Any computer with an Internet connection
  - 1. ❑ Other __________________________________________________

Do your patients access the EHR through the Internet?

- ❑ Yes
  - 1. ❑ No

Is there a mechanism in place to permit several different individuals, using unique passwords, to log­in to a single chart (e.g., to permit patients’ family members to access the EHR)

- ❑ Yes
- ❑ No

Section IV: Perceptions about providing access to the EHR

The following questions are intended to help us understand staff perceptions about the use of the EHR.

Has your hospital conducted any formal survey of staff perceptions around providing patients with access to the EHR?

- ❑ Yes
  1. ❑ No

Would you be willing to provide us with a summary of your findings?

- ❑ Yes
- ❑ No

Please mail or email a summary of your findings to: Dr. Sara Urowitz, Research Associate and Postdoctoral Fellow, Oncology Education and Survivorship, University Health Network Princess Margaret Hospital 14-411610 University Ave. Toronto, ON M5G 2M9 [sara.urowitz@uhn.on.ca](mailto:sara.urowitz@uhn.on.ca)

In your opinion what are the perceptions of staff regarding providing patients with access to the EHR?

- ❑ Open and willing
- ❑ Hesitant but willing
- ❑ Willing to provide partial access
  1. ❑ Other __________________________________________________

Which elements of the EHR are staff most willing to provide patients with access to?

- ❑ The full record
- ❑ Notes
- ❑ Test results
- ❑ Diagnostic Reports
- ❑ Pathology reports
- ❑ Other ________________________________________

The following questions are intended to help us understand patient perceptions about the use of the EHR.

Has your hospital conducted any formal survey of patient perceptions around accessing the EHR?

- ❑ Yes
  1. ❑ No

Would you be willing to provide us with a summary of your findings?

- ❑ Yes
- ❑ No

Please mail or email a summary of your findings to: Dr. Sara Urowitz, Research Associate and Postdoctoral Fellow, Oncology Education and Survivorship, University Health Network Princess Margaret Hospital 14-411610 University Ave. Toronto, ON M5G 2M9 [sara.urowitz@uhn.on.ca](mailto:sara.urowitz@uhn.on.ca)

In your opinion what are the perceptions of patients regarding accessing the EHR?

- ❑ Eager
- ❑ Hesitant but willing
- ❑ Desire only partial access
  - 1. ❑ Other _____________________________________________

Which elements of the EHR are patients most interested in accessing?

- ❑ The full record
- ❑ Notes
- ❑ Test results
- ❑ Diagnostic reports
- ❑ Pathology reports
  - 1. ❑ Other ________________________________________

Is your hospital management supportive of providing patients with access to the EHR?

- ❑ Not Very Supportive
- ❑ Moderately Supportive
  - 1. ❑ Very Supportive

Does your hospital have a champion or steering committee for providing patients with access to the EHR?

- ❑ Yes
- ❑ No
- ❑ Not Sure

Please describe

❑ ___________________________________

There are several barriers that have been identified to effectively giving patients access to the EHR. How important are the following barriers in your hospital?

|  | Not very important |  | Moderately  important |  | Very  important |
| --- | --- | --- | --- | --- | --- |
| Hospital financial resources |  |  |  |  |  |
| Clinician buy-in |  |  |  |  |  |
| Patients’ access to computers |  |  |  |  |  |
| Patients’ computer literacy |  |  |  |  |  |

Placing a high value on embracing change has been identified as a necessary element of providing patients with access to the EHR. How well does your institution manage change?

- ❑ Not Very Readily
- ❑ Moderately
- ❑ Very Readily

It has been suggested that providing patients with access to their EHR should be coupled with aides to better help people understand and cope with the information in the record. The following questions are interested in what type of patient support is provided to individuals who access the EHR?

Does your hospital provide disease-specific education materials?

- ❑ Yes
  - 1. ❑ No

Does your hospital provide general education materials as part of the electronic health record?

- ❑ Yes
  - 1. ❑ No

Does your hospital provide patient education materials specifically designed to help patients understand their EHR?

- ❑ Yes
- ❑ No

Section V: Demographics and Institutional Information

Please indicate your title/position

- ❑ Chief Executive Officer
- ❑ Chief Information Officer
- ❑ Chief of Medicine
- ❑ Chief of Nursing
- ❑ Other ___________________________________

What is the name of your hospital?

❑ ___________________________________

Is your hospital part of a health care system/larger institution?

- ❑ Yes
- ❑ No

What is the name of the health care system/larger institution?

❑ ___________________________________

Where is your hospital/health care system/larger institution located?

- ❑ Alberta
- ❑ British Columbia
- ❑ Manitoba
- ❑ New Brunswick
- ❑ Newfoundland and Labrador
- ❑ Northwest Territories
- ❑ Nova Scotia
- ❑ Nunavut
- ❑ Ontario
- ❑ Prince Edward Island
- ❑ Quebec
- ❑ Saskatchewan
  1. ❑ Yukon

How many beds does your hospital have?

- ❑ Less than 100
- ❑ 100 to 400
- ❑ More than 400
